# Genetic differentiation in the genus *Characodon*: implications for conservation and taxonomy

**DOI:** 10.7717/peerj.11492

**Published:** 2021-07-08

**Authors:** Rosa G. Beltrán-López, Rodolfo Pérez-Rodríguez, Ofelia C. Montañez-García, Juan M. Artigas-Azas, Michael Köck, Adán F. Mar-Silva, Omar Domínguez-Domínguez

**Affiliations:** 1 Laboratorio de Ictiología, Centro de Investigaciones Biológicas, Universidad Autónoma del Estado de Morelos, Cuernavaca, Morelos, México; 2 Programa Institucional de Doctorado en Ciencias Biológicas, Facultad de Biología, Universidad Michoacana de San Nicolás de Hidalgo, Morelia, Michoacán, México; 3 Laboratorio de Biología Acuática, Facultad de Biología, Universidad Michoacana de San Nicolás de Hidalgo, Morelia, Michoacán, México; 4 San Luis Potosí, San Luis Potosí, México; 5 Haus des Meeres Aqua-Terra Zoo, Vienna, Austria

**Keywords:** Goodeidae, Endemic, ESU, Evolutionary history, Taxonomy, Conservation

## Abstract

The subfamily Goodeinae is a group of fishes endemic to the Mexican highlands. Most of the species are restricted to small and isolated streams or springs. Within this subfamily, the genus *Characodon* is the earliest diverging lineage of which three species have been described: *C. lateralis*, *C. audax*, and *C. garmani*, with the latter, considered extinct. *Characodon lateralis* and *C. audax* are classed as endangered, and have been the subject of taxonomic controversy since their description: previous studies have recognized a genetic differentiation in two groups separated by the El Salto waterfall, but morphological analyses contradict these genetic results. We perform a phylogeographic study using the mitochondrial *cytb* gene and *d-loop* region to elucidate the evolutionary history of *C. lateralis* and *C. audax*. The results with both markers show the presence of two highly differentiated haplogroups; one distributed north and the other distributed south of the waterfall, with genetic distances of 1.7 and 13.1% with* cytb* and *d-loop* respectively, and divergence calculated to have occurred 1.41 Mya. Significant genetic structure was found within each haplogroup and suggests the existence of at least four Evolutionary Significant Units (ESUs) within the examined populations. The possible processes identified as contributing to the formation of differentiated genetic groups are isolation, low population size, recurrent bottlenecks, and the strong sexual selection exhibited by the genus.

## Introduction

The members of the subfamily Goodeinae are small-bodied freshwater fishes widespread throughout the Mexican highlands and adjacent areas, encompassing approximately 40 species. Goodeids are notable for their unusual reproductive biology among fishes, typified by internal fertilization, viviparity and matrotrophy (the continuous extra-vitelline supply of nutrients from the parent to the progeny during gestation) (Miller, Minckley & Norris, 2005; see details of these goodeid traits in (Uribe et al., 2018)). The genus *Characodon* is the earliest diverging lineage of the Goodeinae subfamily, sharing a common ancestor with the rest of Goodeinae around 15.5 Mya (Domínguez-Domínguez et al., 2010). The genus is represented by three recognized species: *Characodon lateralis* (Günther, 1866), *C. garmani* (Jordan & Evermann, 1898) and *C. audax* (Smith & Miller, 1986), all of which are restricted to isolated springs and streams in the northern arid highlands of the states of Durango and Coahuila, Northern Mexico (Domínguez-Domínguez, Doadrio & Perez-Ponce De Leon, 2006). The genus has a disjunct distribution pattern: *C. garmani* occurs in the northeast of Mexico within the Nazas River basin (Miller, Minckley & Norris, 2005), whereas *C. audax* and *C. lateralis* occur in the northwest of Mexico at the headwaters of the Mezquital River basin (Domínguez-Domínguez et al., 2010). *Characodon lateralis* was described from an uncertain locality in “Central America”, but this is likely to be an error considering that no specific locality was given for the type material. Previous studies found that the distribution areas of *C. audax* and *C. lateralis* are separated by the waterfall known as El Salto, locating *C. audax* north (above) of waterfall and *C. lateralis* south (below) of it (Domínguez-Domínguez et al., 2010).

Taxonomy and classification of the genus had been historically uncertain and unstable, mainly due to limitations in the original descriptions. *Characodon lateralis* and *C. garmani* were described in 1866 and 1898 respectively, based on a brief description of morphological characters with limited diagnostic value. This led to *C. garmani* being considered synonymous with *C. lateralis* until Smith & Miller (1986) resurrected it as a valid taxon. Likewise, *C. audax* and *C. lateralis* are very similar in morphology, with limited diagnostic traits that differentiate both species (Smith & Miller, 1986). A morphological comparison including eight distinct localities of *C. lateralis* showed that all features considered diagnostic of *C. audax* were found distributed among other populations of *Characodon*, making the two taxa morphologically indistinguishable (Tiedemann & Webb, 2009). A more recent morphological analysis of nine localities from the north and one from the south of the El Salto waterfall found specimens from El Toboso to be distinct from other populations and considered it a valid species (*C. audax*), with further specimens from Cerro Gordo and Los Berros forming distinctive morphological clusters by themselves (Tobler & Bertrand, 2014).

Previous phylogenetic, biogeographic, and phylogeographic studies of goodeids, including several samples of *Characodon* and using mitochondrial and nuclear markers, showed two well-differentiated groups, one distributed north of the El Salto waterfall corresponding to *C. audax* and other distribute south that may correspond to *C. lateralis* (Doadrio & Domínguez, 2004; Howell, Martin & Webb, 2008; Domínguez-Domínguez et al., 2010; Hildreth & Webb, 2012; McCausland & Webb, 2014). However, discussion regarding the appropriate name for each lineage has emerged based on the impossibility of assigning the type locality of *C. lateralis* to a specific water body or even distribution area (Tobler & Bertrand, 2014). Based on bibliographical evidence, it has been argued that the type locality of *C. lateralis* is probably located somewhere south-west of the city of Durango, upstream of the waterfall (Artigas-Azas, 2014), making it an inappropriate name for populations downstream of the waterfall (Lyons et al., 2019).

All three species within the genus are under serious threat (Artigas-Azas, 2002; Artigas-Azas, 2014; Domínguez-Domínguez, Mercado-Silva & Lyons, 2005; Domínguez-Domínguez et al., 2008; Lyons et al., 2019; Lyons et al., 2020). *Characodon garmani* is considered extinct and has not be sighted since the 1890s (Deacon et al., 1979; Smith & Miller, 1986; De la Vega-Salazar, 2006; Domínguez-Domínguez, Doadrio & Perez-Ponce De Leon, 2006). The other two species are also seriously threatened, having been extripated from between 50% and 70% of their historical ranges (Domínguez-Domínguez et al., 2008). Moreover, *Characodon* as a whole inhabits small water bodies in arid northern Mexico that are continuously exposed to extreme abiotic variables and are highly sensitive to any change in habitat. This presents a considerable challenge for the conservation of the genus in light of habitat destruction and climate change. Recent surveys have documented that species of *Characodon* are particularly threatened by the introduction of exotic species, pollution, and the drying out of springs and streams due to groundwater extraction and water diversions (Deacon et al., 1979; Smith & Miller, 1986; De la Vega-Salazar, 2006; Domínguez-Domínguez, Doadrio & Perez-Ponce De Leon, 2006; Lyons et al., 2019).

Knowledge of the genetic variation within and between populations is important for both, taxonomy and conservation. We conduct a phylogeographic study using the mitochondrial cytochrome b gene (*cytb*) and a portion of the control region (*d-loop*), based on extensive sampling of the 11 currently known populations of *Characodon*, and seven of the ten Evolutionary Significant Units (ESUs) described in Lyons et al. (2019). ESUs are populations that are reproductively isolated from other potential ESUs, based upon genetic, morphological, and ecological distinctiveness. ESUs provide a helpful framework for developing protection, restoration, and management plans for wild and captive populations (Ryder, 1986; Waples, 1991; Crandall et al., 2000; Lyons et al., 2019). Phylogenetic analysis allows us to understand the evolutionary history of studied populations, the possible causes of genetic differentiation, and to make taxonomic and conservation interpretations.

## Materials & Methods

### Ethical statement

The care and use of animals complied with SEMARNAT animal welfare laws, guidelines and policies as approved by SEMARNAT-SGA/DGVS/2009/19, SEMACCDET-OS-0084/2019.

### Fish sampling and DNA isolation

**Figure 1:**
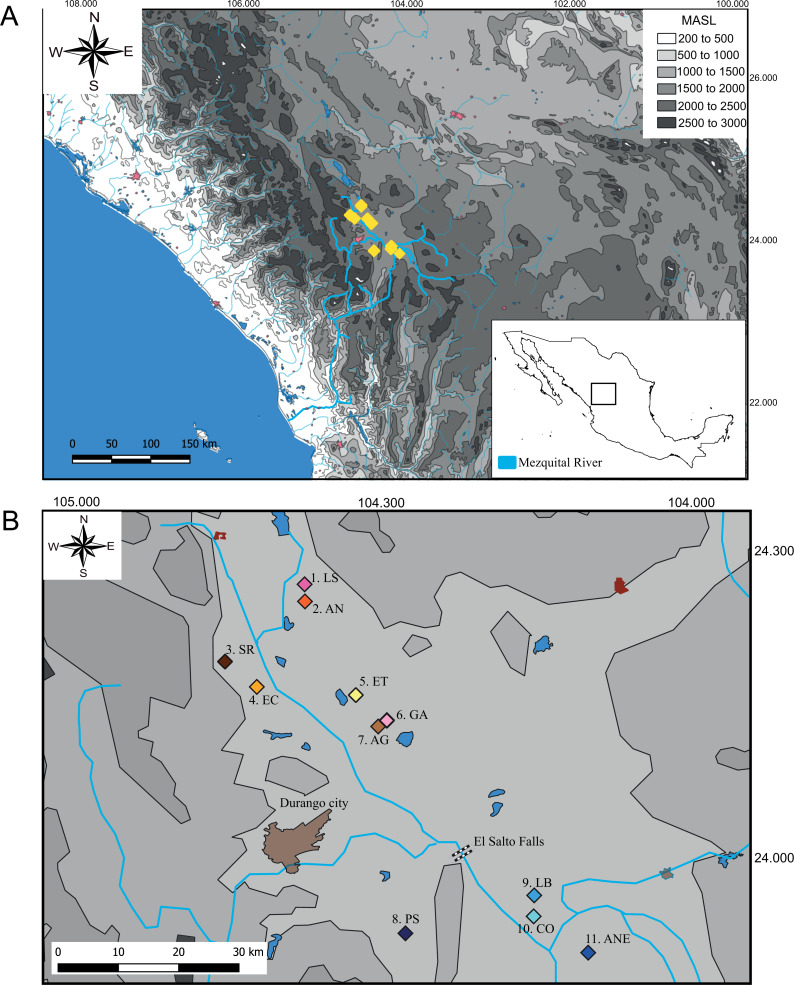
Sampling locations of genus *Characodon*. (A) Topographic map of the Mezquital River showing the elevations of Characodon localities in Meters Above See Level (MASL) (B) Sampling localities along the upper Mezquital River. Numbers correspond to Table 1: 1. Laguna Seca (LS), 2. Anahuac (AN), 3. San Rafael (SR), 4. El Carmen (EC), 5. El Toboso (ET), 6. Garabato (GA), 7. Abraham González (AG), 8. Pino Suárez (PS), 9. Los Berros (LB), 10. Constancia (CO), and 11. Amado Nervo (ANE). Colors of the symbols in sample locations correspond to the colors used in haplotype networks.

We collect 59 specimens in 11 localities across the distribution range of *Characodon* in the Upper Mezquital River (Fig. 1). The samples covered most of the localities where *Characodon lateralis* and *C. audax* have been recorded, including springs, small streams, and stagnant water bodies (Miller, Minckley & Norris, 2005) (Fig. 1; Table 1). Due to taxonomic uncertainties regarding the localities that correspond to *C. lateralis*, throughout the manuscript, we refer to *C. audax* as the El Toboso population and the rest of the samples as populations with the name of the location in which they were sampled (Fig. 1).

**Table 1:** Sampled localities. List of sampled localities with geographic coordinates and number of individuals analyzed for each locality. The numbers and abbreviations in the locality column refer to Fig. 1.

Locality Number	Locality (abbreviation)	*N*	Latitude North	Longitude West
1.	Laguna Seca (LS)	9/14	24°26′6.78″N	104°38′45.6″
2.	Anahuac (AN)	6/5	24°25′16.9″	104°38′19.3″
3.	San Rafael (SR)	5/4	24°19′13.3″	104°46′2.09″
4.	El Carmen (EC)	5/5	24°16′45.7″	104°43′23.4″
5.	El Toboso (ET)	4/5	24°16′29.1″	104°34′54.3″
6.	Garabato (GA)	2/2	24°13′18.6″	104°30′29.3″
7.	Abraham González (AG)	9/7	24°12′53.6″	104°31′47.6″
8.	Pino Suárez (PS)	7/5	23°52′52.7″	104°29′41.1″
9.	Los Berros (LB)	6/3	23°56′20″	104°16′27.7″
10.	Constancia (CO)	5/5	23°54′19.1″	104°16′13.1″
11.	Amado Nervo (ANE)	1/1	23°50′48.4″	104°10′35.9″

**Notes.**

N, sample size for *cytb*/*d-loop*.

We captured fish using electrofishing and seine nets with the permission of the local authorities. We anesthetized all specimens with tricaine-mesylate (MS-222). We obtain pectoral fin clips and preserved tissues in absolute ethanol, frozen at −75 °C, and deposited in the tissue collection of the Laboratorio de Biologia Acuatica of the Universidad Michoacana de San Nicolas de Hidalgo. We returned all specimens to the water following tissue extraction. We extracted DNA by digesting tissues with Buffer ATL (QIAGEN) and Proteinase K and purifiying using the BioSprint DNA Blood Kit (QIAGEN) according to the manufacturer’s instructions.

### Locus amplification and sequencing

We amplified fragments of two portions of the mitochondrial genome, the cytochrome b gene (*cytb*) and a portion of the control region (*d-loop*), via polymerase chain reaction (PCR) using samples from 59 and 56 individuals respectively. We used the primers HA and LA (Dowling et al., 2002) to amplify *cytb*. We used the primers Dloop-A and Dloop-E (Lee et al., 1995) to amplify *d-loop*. We performed the PCR reaction in a final volume of 12.5 µl, containing 4.25 µl of nuclease-free water, 0.5 µl of each 0.2 *μ*M primer, 6.25 µl Dream *Taq* Green PCR Master Mix 2x containing DreamTaq DNA polymerase, 2X Dream Taq Green buffer, dATP, dCTP, dGTP and dTTP, 0.4 mM each, and 4 mM MgCl_2_ (Thermo Scientific, Waltham, MA, USA), and 1 µl (ca. 10-100 ng) of DNA template. The PCR procedure is described in Table S1. We purified the amplicons using ExoSAP-IT (USB Corp. Cleveland, OH, USA) and submitted to Macrogen Inc. (Korea) for sequencing. We implemented a manual alignment of the sequences in Mega v10.1.7 (Kumar et al., 2018). We deposited only the sequences of the different haplotypes in GenBank under the accession numbers (*cytb*: MW208628–MW208647 and, *d-loop*: MW208648–MW208661) (see Table S2).

### Haplotype networks

To evaluate the geographic correspondence of haplotype distribution, we conducted a phylogenetic network estimation for each gene using the median-joining algorithm, using the default parameters as implemented in PopArt v1.7. (http://popart.otago.ac.nz/index.shtml).

### Time-calibrated species-tree

We collapsed to haplotypes the DNA sequences of each of the two markers (*cytb* and *d-loop*) using the web-based program ALTER (González-Peña et al., 2010). We used alignments for each marker to estimate and select the substitution model that best fitted the datasets, using the Akaike information criterion and partition settings performed in PartitionFinder v1.1.0 (Lanfear et al., 2012). We obtaining the optimal partition setting by assigning one substitution model to each gene, the same model for both genes: GTR+I. We performed a time-calibrated species-tree analysis under a multispecies coalescent model using *BEAST v.1.8.1 (Heled & Drummond, 2010). We tested three different hypotheses based on (1) Previous genetic studies in which were established the differentiation between populations (Domínguez-Domínguez et al., 2010), (2) considering the ESUs recovered by Lyons et al. (2019) based on genetic, morphology, and zoogeography information, and (3) according to our haplotype networks and genetic differentiation results. The first hypothesis (H1) assumes the independence of each recovered haplogroup of *Characodon* in the haplotype network, in which all northern populations (NP) (north of the El Salto waterfall: Laguna Seca, San Rafael, Anahuac, El Carmen, El Garabato, Pino Suarez, Abraham Gonzalez and *C. audax*) were grouped and the southern populations (SP) (south of the El Salto waterfall: La Constancia, Los Berros and Amado Nervo) were grouped. In the second hypothesis (H2) we considered *C. audax*, the rest of the northern populations (RNP), and all SP (La Constancia, Los Berros, and Amado Nervo) as independent groups, based upon previous genetic studies (Doadrio & Domínguez, 2004; Domínguez-Domínguez et al., 2010; Lyons et al., 2019; Tobler & Bertrand, 2014). In the third hypothesis (H3) we considered *C. audax*, the RNP, La Constancia + Los Berros, and Amado Nervo as independent groups (based upon the results of this study).

We applied a lognormal relaxed clock (uncorrelated) model on branch length and calibrated the *cytb* partition using the mutation rate in teleosts of 0.76–2.2%/million years (Zardoya & Doadrio, 1999; Machordom & Doadrio, 2001; Near & Benard, 2004). We estimated the evolutionary rate of the *d-loop* relative to the *cytb* gene, as has been implemented for other fish species (Perea, Cobo-Simo & Doadrio, 2016; Beltrán-López et al., 2017; Sandoval-Huerta et al., 2019). Given the high divergences found between populations, we used the tree prior Yule Process model (Gernhard, 2008) and estimated a starting tree using the random method. We ran Markov Chain Monte Carlo analysis for 100 million generations, sampled every 500 generations. We evaluated chain convergence with the -InL values in Tracer v.1.5 (Rambaut & Drummond, 2007) and summarized the results using TreeAnnotator v.1.8.1 (Drummond et al., 2012).

### Genetic distances and structure

To quantify the genetic differences between assumed genetic groups or populations, we calculated the uncorrected *p-* distances for both genes considering two different arrangements according to the results obtained in the species tree analyses: H1) the two recovered haplogroups of *Characodon* in the haplotype network, in which all northern populations (NP) were grouped and all southern populations (SP) were grouped*,* and H3) *C. audax* as a differentiated group (population from El Toboso within NP), with the rest of the NP as another group, and SP samples grouped as: La Constancia + Los Berros as a single group and Amado Nervo as an independent group.

To analyze the genetic structure of populations of *Characodon*, we conduct an analysis of molecular variance (AMOVA) using 10,000 permutations in Arlequin v3.5.1.3 (Excoffier & Lischer, 2010) at the same two hierarchical levels tested for the *p-* distances analyses. We also calculated components of the fixation index Φ_*CT*_, Φ_*ST*_ and Φ_*SC*_ using Arlequin v3.5.1.3 (Excoffier & Lischer, 2010). We estimated genetic differentiation among populations with paired test fixation indices (Φ_*ST*_) for each gene, under the same scenarios used in genetic distances and AMOVA analyses. We applied a Bonferroni correction (Rice, 1989) to each *p*-value obtained in the paired test of genetic differentiation.

### Genetic diversity

We estimated the levels of genetic diversity, including the number of haplotypes (H), polymorphic sites (*S*), nucleotide diversity (*π*) and haplotype diversity (*h*) for each gene in Arlequin v3.5.1 (Excoffier & Lischer, 2010) under the same two arrangements used for genetic distances, AMOVAs and paired test fixation indices. The population from Amado Nervo was excluded since only one sequence was available from that source.

### Species delimitation

We performed species delimitation analyses and species tree estimation in the program BPP v3.4 (Flouri et al., 2018). The method uses the multispecies coalescent model to compare different models of species delimitation (Yang & Rannala, 2014; Rannala & Yang, 2017). These analyses were based on the two mitochondrial markers studied (*cytb* and *d-loop*). We implemented all species delimitation analyses under three hypotheses, in the same manner as the time-calibrated species-tree. We implemented the A10 analysis (species delimitation using a fixed guide tree) (Yang & Rannala, 2010) using the relationship obtained from the time-calibrated species-tree as a guide tree for each tested hypothesis. We assigned the population size parameters (*θ*s) the inverse-gamma prior IG (3, 0.002), with mean 0.002/(3-1) = 0.001 (Flouri et al., 2018). We assigned the divergence time at the root of the species tree (*τ*0) the inverse-gamma prior IG (3, 0.004), with a mean 0.002, while the other divergence time parameters were specified by the uniform Dirichlet distribution (Yang & Rannala, 2010: Eq. 2). We set the MCMC to 200,000 samples with burn-in = 20,000 and sample frequency = 2. We assessed convergence by comparing the consistency of the posterior distributions. We ran each analysis at least twice to confirm consistency between runs. To accommodate uncertainty in the guide tree, we performed the analysis A11 (joint species delimitation and species-tree estimation) using the same prior sets and tested the same hypothesis as in the A10 analysis.

## Results

### Samples and sequence data

We obtained 59 sequences of the *cytb* mitochondrial gene (1081 base pairs (bp)) and 56 sequences of the *d-loop* (384 bp) distributed in 11 localities across the distribution range of *Characodon* in the Mezquital basin (Table 1 and Fig. 1). The Amado Nervo population is considered to have been extirpated since at least 2005 (Lyons et al., 2019), and only one sequence was obtained from museum specimens. Of the 1,081 *cytb* bp, 30 sites were polymorphic, 24 were parsimony informative, and 6 were singleton variable sites. For the *d-loop*, of the 384 bp, 53 sites were polymorphic, 48 were parsimony informative, and 5 were singleton variable sites.

### Haplotype networks

**Figure 2:**
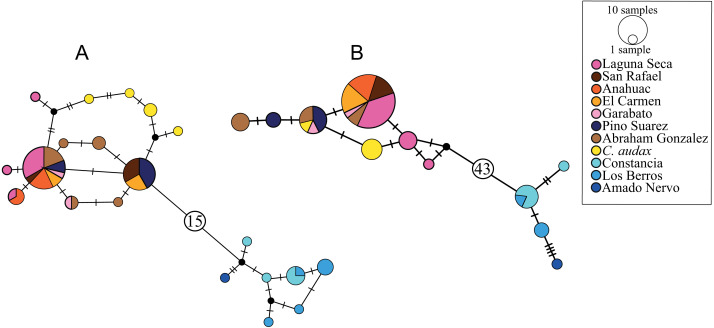
Haplotype networks. (A) Based on mitochondrial gene *cytb*, (B) based on mitochondrial *d-loop* of the control region. The size of the circles indicates the frequency of the haplotype. The colors correspond to those used in location symbols as shown in Fig. 1. The dash between haplotypes corresponds to the mutational steps. The small black circles correspond to hypothetical haplotypes, and the numbers inside the circles (A) 15 and (B) 43 indicate the number of mutational steps between haplogroups.

The haplotype network for the *cytb* marker recovered 20 haplotypes, with a clear structure in two haplogroups (Fig. 2A). One of these haplogroups includes all of the samples north (NP) of El Salto waterfalls, with one central haplotype that includes the samples from all localities, except for *C. audax*, which does not share haplotypes and is separated at least by two mutation steps from the rest of the northern samples (RNP; Fig. 2A). The NP group is separated by 16 mutation steps from another haplogroup formed by the samples south of the waterfall (SP; Fig. 2A). In this haplogroup, three different localities are included (La Constancia, Los Berros, and Amado Nervo). Los Berros and La Constancia share one haplotype. The only sample from Amado Nervo is separated by four mutation steps from other SP samples (Fig. 2A). The same arrangement of two haplogroups, SP and NP, was found with the *d-loop* marker, but separated by 44 mutation steps (Fig. 2B). Most of the *C. audax* samples fall in a unique haplogroup, except for one that is shared with other NP samples. We found shared and unique haplotypes from La Constancia and Los Berros, whereas the only sample included from Amado Nervo is separated by four mutation steps from the rest of the SP samples (Fig. 2B).

### Time-calibrated species-tree

**Figure 3:**
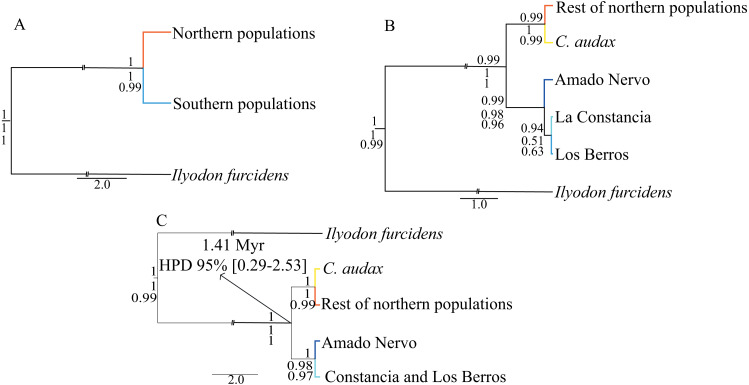
Time-calibrated species-tree and species delimitation. Time-Calibrated Species-Tree with both concatenated genes, (A) hypothesis H1, considering the two recovered haplogroups (NP and SP) as putative species; (B) hypothesis H2, considering five putative species: *C. audax*, RNP, Constancia, Berros, and Amado Nervo; and (C) hypothesis H3, considering four putative species: *C. audax*, RNP, Amado Nervo, and Constancia+Berros. The posterior probabilities of the Time-Calibrated Species-Tree are shown above the branches. The probabilities of species delimitation test of A10, followed by the A11 analyses are shown below the branches. The divergence time estimations with 95% High Posterior Density (HPD) are shown in the nodes of the hypothesis H3.

For the time-calibrated species-tree analyses (Fig. 3), the H1 arrangement of two putative species (NP and SP) was highly supported, showing posterior probabilities (Pp) of 1.0 (Fig. 3). For the H2 arrangement of five putative species, the lowest support was for the relationship between La Constancia and Los Berros (Pp = 0.94), the rest of the relationships in this arrangement were supported with Pp>0.99. Whereas the H3 arrangement of four putative species (*C. audax*, RNP, Amado Nervo, and La Constancia + Los Berros) was highly supported, with Pp = 1.0 for all relationships (Fig. 3).

The mutation rate-calibrated tree suggests that the first isolation event that separated NP and SP probably occurred during the Pleistocene *ca*. 1.41 Mya (95% HPD: 0.29−2.53 Mya) (Fig. 3).

### Genetic distances

The uncorrected genetic distances between the two haplogroups (NP and SP) were 1.7% and 13.1% for *cytb* and *d-loop* respectively. When genetic distances were calculated for four groups: (1) *C. audax*, (2) the RNP as an independent group, (3) La Constancia + Los Berros, and 4) Amado Nervo, the values for *cytb* range from 0.3% between *C. audax* and RNP to 1.8% between *C. audax* and La Constancia + Los Berros and *C. audax* and Amado Nervo. The genetic distances for *d-loop* range from 0.5% between *C. audax* and RNP, to 13.3% between RNP and Amado Nervo, and between *C. audax* and Amado Nervo (Table 2).

**Table 2:** Uncorrected *p* genetic distances. Uncorrected *p* genetic distances in percentage values between groups (below diagonal) and within groups (in the diagonal). Pairwise Φ_ST_ (above diagonal). Significant values after Bonferroni correction (*p* < 0.05) are presented in bold.

***cytb***					
		Northern populations	Southern populations		
H1	Northern populations	0.1%	**0.911**		
	Southern populations	1.7%	0.2%		
		Rest of the northern populations	*C. audax*	Amado Nervo	Berros and Constancia
H3	Rest of the northern populations	0.1%	**0.637**	0.949	**0.939**
	*C. audax*	0.3%	0.1%	0.922	**0.913**
	Amado Nervo	1.7%	1.8%	n/c	0.591
	La Constancia + Los Berros	1.7%	1.8%	0.4%	0.2%
***d-loop***					
		Northern populations	Southern populations		
H1	Northern populations	0.3%	**0.959**		
	Southern populations	13.1%	0.5%		
		Rest of the northern populations	*C. audax*	Amado Nervo	Berros and Constancia
H3	Rest of the northern populations	0.3%	**0.476**	0.981	**0.967**
	*C. audax*	0.5%	0.1%	0.993	**0.927**
	Amado Nervo	13.3%	13.3%	n/c	**0.524**
	La Constancia + Los Berros	12.6%	12.6%	1.4%	0.2%

### Genetic structure among populations

For *cytb*, the AMOVA arrangements showed Φ_ST_ values of 0.92 for four groups and 0.94 for two groups, with Φ_SC_ values of 0.22 for the four groups and 0.39 for two groups, and Φ_CT_ values for both arrangements of 0.90, with all fixation indices showing significant results (*p*  < 0.05). For *d-loop*, the Φ_ST_ values were 0.96 and 0.97 for four and two groups, respectively. The Φ_SC_ values were 0.28 and 0.43 for four and two groups, and the Φ_CT_value was 0.95 for both arrangements, with all fixation indices showing significant results (*p* <  0.05) (Table 3).

**Table 3:** Analyses of molecular variance. Analyses of molecular variance for two grouping schemes and both genes. All fixation index values were significant ( *P* < 0.05).

***cytb***			
Testing assumptions	Source of variation	% of variance	Fixation index
H1) Southern populations / Northern populations	Between groups Between populations within groups Within populations Total	90.59 3.72 5.67 100	Φ_CT_: 0.90 Φ_SC_: 0.39 Φ_ST_: 0.94
H3) *C. audax* / rest of the northern populations / La Constancia + Los Berros / Amado Nervo	Between groups Between populations within groups Within populations Total	90.48 2.12 7.40 100	Φ_CT_: 0.90 Φ_SC_: 0.22 Φ_ST_: 0.92
***d-loop***			
H1) Southern populations / Northern populations	Between groups Between populations within groups Within populations Total	95.72 1.85 2.43 100	Φ_CT_: 0.95 Φ_SC_: 0.43 Φ_ST_: 0.97
H3) *C. audax* / rest of the northern populations / La Constancia + Los Berros / Amado Nervo	Between groups Between populations within groups Within populations Total	95.05 1.40 3.56 100	Φ_CT_: 0.95 Φ_SC_: 0.28 Φ_ST_: 0.96

The pairwise Φ_ST_ values for *cytb* and *d-loop* for NP and SP showed high and significant genetic differentiation (>0.90) (Table 2). When the arrangement was based on four groups, the genetic differentiation for most comparisons was high, although not all were significant. The lowest genetic differentiation for both genes was the comparisons between groups within the SP (Table 2).

### Genetic diversity

The genetic diversity for the two main recovered haplogroups showed higher haplotype diversity in SP for both genes (0.863 for *cytb* and 0.955 for *d-loop*), while for the NP the haplotype diversity was 0.760 for *cytb* and 0.659 for *d-loop* (Table 4).

**Table 4:** Genetic diversity for the genus *Characodon*. Genetic diversity for two grouping schemes: (1) the two recovered haplogroups NP and SP, and (2) considering *C. audax*, the RNP, La Constancia + Los Berros together, and Amado Nervo as independent groups.

	**N**	**S**	**H**	*π*	***h***
***cytb***					
H1) Northern populations	49	11	13	0.001+/-0.000	0.760+/-0.049
Southern populations	12	8	7	0.00+/-0.001	0.863+/-0.078
***d-loop***					
H1) Northern populations	49	7	6	0.003+/-0.002	0.659+/-0.064
Southern populations	10	23	8	0.021+/-0.012	0.955+/-0.059
***cytb***					
H3) Rest of the northern populations	43	5	8	0.000+/-0.000	0.688+/-0.055
*C. audax*	4	3	3	0.001+/-0.001	0.833+/-0.222
La Constancia + Los Berros	11	6	6	0.001+/-0.001	0.836+/-0.088
***d-loop***					
H3) Rest of the northern populations	42	6	6	0.002+/-0.002	0.566+/-0.082
*C. audax*	5	1	2	0.001+/-0.001	0.400+/-0.237
La Constancia + Los Berros	8	19	6	0.017+/0.010	0.928+/-0.084


**Notes.**

N sample size S polymorphic sites H number of haplotypes *π* nucleotide diversity h haplotype diversity

When the genetic diversity was calculated considering four groups, the highest genetic diversity for both genes was for La Constancia + Los Berros (*cytb; π* = 0.001 and *h* = 0.836 and *d-loop; π* = 0.017 and *h* = 0.928) (Table 4).

### Species delimitation

The species delimitation for the A10 and A11 analyses produced similar results, which indicated that the estimated species tree did not differ from the inferred time-calibrated species-tree. Both the A10 and A11 analyses strongly support hypothesis H1 of two putative species (NP and SP as different species), and hypothesis H3 of four putative species (*C. audax*, rest of Northern populations, Amado Nervo, and La Constancia + Los Berros) (Fig. 3).

## Discussion

### Split of north and south clades

All of the analyses presented in this study support the recognition of two well-differentiated groups (Figs. 2 and 3, Tables 2 and 3): one distributed south and another distributed north of the El Salto waterfall. The molecular clock shows that this isolation event occurred at *ca*. 1.41 Mya (Fig. 3). Our results support previous studies that showed the split of these two groups occurred around 1.5 to 2.2 Mya, describing the isolation as a vicariant event promoted by the appearance of El Salto waterfall (Domínguez-Domínguez, Doadrio & Perez-Ponce De Leon, 2006; Domínguez-Domínguez et al., 2010). This is consistent with the eruptions of lava that occurred in the late Pliocene or early Pleistocene on the Mezquital River that gave rise to a fall of more than 30 m (Albritton, 1958). The closest populations between NP and SP are separated by a distance of around 60 km following the course of the river, but show a high genetic distance of 1.7% and 13.1% with the *cytb* and *d-loop* respectively (Table 2), whereas the two most distant populations that share haplotypes within NP (Pino Suarez and Laguna Seca) are separated by approximately 80 km following the course of the river. This supports the notion that the waterfall, and not the distance, constitutes a biogeographic barrier that isolates the two genetic groups (Fig. 1B).

The Mezquital River has a high level of fish endemism, with endemic species such as *Chirostoma mezquital*, *Cyprinodon meeki*, *Notropis aulidion,* and *Moxostoma milleri* (Meek, 1904; Miller, 1976; Chernoff & Miller, 1986; Pérez-Rodríguez et al., 2016, respectively). Previous genetic studies have also documented several undescribed and possible endemic species within the drainage, as is the case of *Ictalurus* sp, *Dionda* sp, *Gila* sp, *Codoma* sp, and *Pantosteus* sp (Lundberg, 1992; Schönhuth et al., 2012; Schönhuth et al., 2014a; Schönhuth et al., 2014b; Corona-Santiago et al., 2018, respectively). These cases highlight the importance of Mezquital River as a center of endemism. This is the first study to examining genetic structure at the population level, based on a phylogeographic approach, in a fish species along the Mezquital River.

### Population structure

#### North clade

Our results also show the formation of well-differentiated groups within each genetic group. For the NP, *C. audax* (El Toboso population) shows significant genetic differentiation (Tables 2 and 3; Fig. 2), with almost all haplotypes being exclusive, but with low genetic distances of 0.3% and 0.5% in *cytb* and *d-loop* respectively. El Toboso, the locality to which *C. audax* is supposed to be endemic, is located at a distance of 7 km from the nearest population of other *Characodon*, and less than 50 m from the Rio de la Sauceda, the northern tributary of the Mezquital River. El Toboso is represented by an isolated spring that intermittently flows through a volcanic rock bed to the ephemeral and seemingly endorheic Lake El Toboso (Miller & Smith, 1986). The spring and the Lake have dried up several times through history: During March 1982, April 1983, and May 1985 (Miller & Smith, 1986), and in April 1999, 2003, and 2011 according to our field observations. The genetic distinctiveness of *C. audax* could be the result of restricted gene flow among populations due to geographic isolation, local adaptation to a particular habitat (the blackish coloration of the body in *C. audax*, which appears to be an adaptation to blend in with the volcanic rock bottom of its habitat), genetic drift due to recurrent bottlenecks induced by the drying events, and the strong sexual selection that occurs in the species, as has been suggested before to explain morphological differences in *Characodon* populations (Tobler & Bertrand, 2014). Previous studies also support the notion that the higher rate of evolution in the Goodeinae could be related to the vicariance promoted by the fragmentation of river basins, the ecological opportunities generated by modification in river basins, and the advantages provided by viviparity, in conjunction with sexual selection (Pérez-Rodríguez et al., 2015; Foster & Piller, 2018). Also, within the Goodeinae, higher levels of population differentiation (*F*_ST_) has been found in the more dimorphic species, implying lower gene flow between populations, as could be happening in *Characodon* (Ritchie et al., 2007).

#### South clade

The three studied populations within the SP also show genetic differentiation (Figs. 2 and 3; Tables 2 and 3). The sample from the Amado Nervo population displayed the highest genetic distance within the South clade, even higher than that recorded between *C. audax* and the rest on northern populations (Table 2). However, it is important to point out that the inferences made in the Amado Nervo population were based on a single specimen and therefore these results should be interpreted with caution. To further investigate the distinctiveness of the Amado Nervo population future studies should seek to increase the sample size (with museum specimens) or the use of other molecular markers (such as SNPs) to evaluate the distinctiveness of the population. Previous studies have also found high genetic differentiation (Domínguez-Domínguez et al., 2010) and coloration distinctiveness (Tobler & Bertrand, 2014) of the Amado Nervo population when compared to other SP. The Amado Nervo population is historically recognized to be a small population that inhabits a little spring and a segment of a tributary (the Amando Nervo). The population has not been seen since 2005 (Lyons et al., 2019). Although no information is available for this population, it is probable that, as with *C. audax*, the high genetic divergence displayed by both molecular markers could be related to genetic drift or the interaction of the latter with other evolutionary drivers, such as isolation or selection.

### Taxonomic implications

The formation of two highly divergent NP *vs* SP clades (1.7% and 13.1% in *cytb* and *d-loop* respectively) showen in the present study corroborates previous studies using mitochondrial (Doadrio & Domínguez, 2004; Domínguez-Domínguez et al., 2010) and nuclear markers (Hildreth & Webb, 2012; McCausland & Webb, 2014). The genetic distances between NP and SP obtained herein were similar to those found between recognized species of goodeids based on the *cytb* gene (Doadrio & Domínguez, 2004; Corona-Santiago, Doadrio & Domínguez-Domínguez, 2015; Beltrán-López et al., 2017), whereas the distances for the *d-loop* were higher than those obtained between species of the genus *Ilyodon*, belonging to the Goodeidae family (Beltrán-López et al., 2017).

As previously mentioned, the taxonomy of the *Characodon* species is complex (Tobler & Bertrand, 2014; Lyons et al., 2019). The type locality of *C. lateralis* is unknown, however, through a bibliographic revision, we found that it is highly likely that *C. lateralis* type material was collected above the El Salto waterfall. The specimens used as type material were collected by Dr. Berthold Carl Seemann in Durango (mistakenly assigned to Central America by Albert Günther). Smith & Miller (1986) recognized that their material for *C. lateralis*, collected in the upper stretches of the Rio Mezquital, correspond to the same species that Günther used in his description in 1866. The collector of the type material, Dr. Berthold Seemann, was appointed as a naturalist on the voyage of exploration of the American west coast and Pacific on the HMS Herald, 1847–1851. As such, he visited Durango during December 1848 and February 1849. His detailed account is given in his Narrative of the voyage of H.M.S. Herald (Seeman, 1853:159). In his memoirs, he recognizes a single exploration southwest of Durango by the road to Tepic, passing by habitats of *C. lateralis* above the El Salto waterfall, particularly Puente Pino Suarez. He also crossed the Mezquital River at Mezquital; however, at that point along the river there are no historical populations of *C*. *lateralis*. This indicates that the types could have been collected above the El Salto waterfall. Given the detailed account of Dr. Seemann, it seems unlikely that he would have overlooked the impressive falls if he had visited them. All this evidence suggests that the species *C. audax* and *C. lateralis* were described from specimens north of the El Salto waterfall, but the exact location of the type material for *C. lateralis* remains unknown.

The results presented herein for the northern populations shows *C. audax* from El Toboso is significantly genetically differentiated from the rest of the northern populations (Laguna Seca, San Rafael, Anahuac, El Carmen, El Garabato, Pino Suarez, and Abraham Gonzalez) (Figs. 2 and 3, Tables 2 and 3), but the uncorrected *p* genetic distances were much lower (0.3% for *cytb* and 0.5% for *d-loop*) than the divergences found between recognized species within Goodeidae (1.7% for *cytb* gene; (Doadrio & Domínguez, 2004; Corona-Santiago, Doadrio & Domínguez-Domínguez, 2015; Beltrán-López et al., 2017). In the original description by Smith & Miller (1986), the 32 specimens of *C. lateralis* and 28 of *C. audax* analyzed showed very similar body shape and the measurements formed clusters that overlapped broadly, with the only differentiation being the shape of the dorsal profile. This difference of shape of the dorsal profile, was however not recovered in the morphometric analysis made by the same authors, where males of both species can usually be distinguished by the position of the anus and pelvic fins. A recent morphological analysis of eight localities, including the type material of *C. lateralis*, concluded that the analysis by Smith & Miller (1986) was likely to have been flawed, with nearly all features considered diagnostic of *C. audax* found distributed among other populations of *Characodon*, making them morphologically indistinguishable from one another (Tiedemann & Webb, 2009). Whereas, the study conducted by Tobler & Bertrand (2014), which includes 530 individuals in ten locations covering the entire distribution range of the genus, concludes that *C. audax* (El Toboso population) exhibits the most divergent body shape, differing significantly from all other investigated populations of the NP, with the variation mainly related to body height, proportions of the caudal peduncle, head shape, and the length of the anal and dorsal fin bases.

In the case of the SP, the genetic structure results show significant differences among La Constancia, Los Berros, and Amado Nervo (SP group) (Tables 2 and 3), indicating that these three populations may represent independent evolutionary lineages. However, the species tree and BPP analyses do not support the segregation of La Constancia and Los Berros (Fig. 3), and both populations shared haplotypes (Fig. 2). According to our results, we consider that both Los Berros + La Constancia form a unique lineage, separated from Amado Nervo. These results were also supported by the species tree analysis (Fig. 3). These two genetically differentiated groups show uncorrected *p* genetic distances (*cytb* >0.4% and *d-loop* >1.4%) that are higher than the differences between *C. audax* and other populations within NP. However, due to the low sample size from Amado Nervo and the use of exclusively mitochondrial markers, these results should be viewed with caution. We recommend that further research should be carried out on the Amado Nervo population utilising other molecular markers and SNPs to clarified the genetic structure and taxonomy of the genus. Our results also support the morphological study of Tobler & Bertrand (2014), in which the Los Berros population (the only population investigated that occurs south of the El Salto waterfall) was found to have a divergent body shape and to be differentiated from other populations.

According to the results presented herein, we suggest the existence of two well-differentiated and putative species, one north and the other south of the El Salto Waterfall. In the case of *C. audax*, the genetic result presented herein and the contradictory results in morphological evidence prevent any conclusion about its taxonomic status. We consider *C. audax* to be a valid species pending a more extensive and integrative taxonomic study. According to our results, if *C. audax* is confirmed to be endemic to El Toboso, the rest of the Northern populations would have to be considered as *C. lateralis*, whereas the SP emerges as an undescribed taxon. All the above highlight the necessity for an extensive integrative taxonomic study to elucidate the taxonomic status of the genetically and morphologically differentiated groups.

### Conservation implications

We found a generally high genetic diversity within populations of *Characodon*, with *h* >0.7 for *cytb*, and *h* >0.4 for *d-loop* (Table 4) although, in some cases, the sample size was low (e.g., Los Berros, *N* = 3). The high genetic diversity contrasts with the decline in population size and the low population estimation for species living in springs arising from desert areas, indicating that the populations seem to maintain diversity despite their depauperate nature (Lyons et al., 2019). Although we found only four genetically differentiated groups in our analyses that include seven of the ten ESUs described by Lyons et al. (2019) for the genus, we agree with the ESUs designation of Lyons et al. (2019), since they use not only genetic information but also include morphological and zoogeographic considerations. We recommend that a wider sampling effort and the inclusion of more molecular markers is needed to further develop the understanding of the conservation genetics of this highly threatened fish genus.

Populations of *Characodon* have shrunk dramatically due to the pressures on aquatic habitats in the arid regions of the Mexican Plateau. The conservation problems facing *Characodon* species have been noted since their declaration as threatened by Deacon et al. (1979). Smith & Miller (1986) stated that *C. lateralis* had disappeared from Rio Tunal south of Durango City by 1968, where the species had been common in 1963 (Contreras-Balderas, 1975). Several studies reporting the decline in the abundance and distribution of populations of *Characodon* have been conducted more recently (De la Vega-Salazar, 2006; Artigas-Azas, 2014; Lyons et al., 2020), reporting the disappearance of more than 40% of the historically known populations of *Characodon* (Domínguez-Domínguez et al., 2008) and the recent extinction of three ESUs (Lyons et al., 2019). Some of the factors responsible for the depletion of these species include pollution, habitat destruction, water overexploitation, and introduction of non-native fish (Lyons et al., 2019). Such anthropogenic perturbations have been found to be more destructive in desert areas (Vale & Brito, 2015) such as those occupied by the *Characodon* genus. The results of this study should be used to inform conservation priorities in targeting populations of *Charadon* for conservation intervention to tackle declines in the genus.

## Conclusions

We recognized two well-differentiated and highly divergent genetic and geographic groups within the genus *Characodon*, one distributed to the north (NP) and the other distributed to the south (SP) of the El Salto waterfall. We suggest that this waterfall acts as a biogeographic barrier that isolated the two groups at *ca.* 1.41 Mya. We also found genetic differentiation within each group. In NP the species *C. audax*, endemic to the El Toboso watercourse, do not share haplotypes with the rest of the populations and show significant genetic differentiation, although the genetic divergences are low. Within the SP, the three studied populations show genetic differentiation, concordant with morphological differentiation observed in previous studies (Tobler & Bertrand, 2014). The taxonomy of the genus is complex. The type locality of *C. lateralis* is unknown, and recent morphological studies were unable to distinguish differences between populations. This highlights the necessity for an extensive integrative taxonomic study. Regardless of taxonomic uncertainty, the conservation of extant populations of *Characodon* is urgently needed.

##  Supplemental Information

10.7717/peerj.11492/supp-1 Supplemental Information 1 PCR procedures and GenBank access number PCR procedures for each genes is shown in the first table, also the primers used for each genes, in the second table are the specimen information and, also de genbank access number

## Additional Information and Declarations

### Data Availability

The following information was supplied regarding data availability: The data is available at figshare: Beltrán López, Rosa (2021): Concatenados. fasCharacodon concatenados. figshare. Dataset. https://doi.org/10.6084/m9.figshare.13167950.v1. MW208628 to MW208647 and *d-loop*: MW208648–MW208661.
