# Supplementary material for: Genetic differentiation in the genus Characodon: implications for conservation and taxonomy
Source: PeerJ. 2021 Jul 8;9:e11492. doi: 10.7717/peerj.11492 (PMC8272924; doi:10.7717/peerj.11492)
Supplement: Supplemental Information 1 — PCR procedures for each genes is shown in the first table, also the primers used for each genes, in the second table are the specimen information and, also de genbank access number [file peerj-09-11492-s001.doc]

Table S1 PCR procedure

|  | *cytb* | *d-loop* |
| --- | --- | --- |
| Primers | HA  LA | Dloop-A  Dloop-E |
| Size (bp) | 1081 | 384 |
| Reference | Dowling et al., (2002) | Lee et al., (1995) |
| Denaturing (step 1) | 94 ºC, 5 min. | 95 ºC, 1 min. |
| Cycles (step 2) | 35 | 32 |
| Denaturing | 94 ºC, 45 s. | 94 ºC, 30 s. |
| Annealing | 48 ºC, 60 s. | 48 ºC, 30 s. |
| Extension | 72 ºC, 90 s. | 72 ºC, 60 s. |
| Final extension (step 3) | 72 ºC, 10 min. | 72 ºC, 10 min. |

Table S2 Specimen information and GenBank accession numbers

| Site | Locality | Biogeographic region | Voucher number | GenBank access number  *cytb d-loop* | |
| --- | --- | --- | --- | --- | --- |
| 1 | Laguna Seca | Mezquital River | 3688 | MW208628 | NO |
| 1 |  |  | 4982 | NO | MW208648 |
| 1 |  |  | 28365 | MW208629 | NO |
| 1 |  |  | 28369 | NO | MW208649 |
| 1 |  |  | 28371 | NO | MW208650 |
| 1 |  |  | 28375 | MW208630 | NO |
| 5 | El Toboso |  | 3681 | MW208631 | NO |
| 5 |  |  | 28414 | MW208632 | NO |
| 5 |  |  | 28415 | MW208633 | MW208651 |
| 5 |  |  | 28418 | MW208634 | NO |
| 7 | Abraham González |  | 1 | MW208635 | NO |
| 7 |  |  | 28409 | MW208636 | MW208652 |
| 7 |  |  | 28410 | MW208637 | NO |
| 7 |  |  | 28411 | MW208638 | NO |
| 7 |  |  | 28412 | MW208639 | NO |
| 8 | Pino Suárez |  | 4963 | NO | MW208653 |
| 8 |  |  | 4964 | MW208640 | MW208654 |
| 9 | Los Berros |  | 28431 | MW208641 | MW208655 |
| 9 |  |  | 28432 | MW208642 | MW208656 |
| 9 |  |  | 28433 | MW208643 | NO |
| 10 | Constancia |  | 28426 | MW208644 | MW208657 |
| 10 |  |  | 28427 | MW208645 | MW208658 |
| 10 |  |  | 28428 | NO | MW208659 |
| 10 |  |  | 28430 | MW208646 | MW208660 |
| 11 | Amado Nervo |  | 8864 | MW208647 | MW208661 |
